# Antimicrobial and Antioxidant Properties of Total Polyphenols of *Anchusa italica* Retz

**DOI:** 10.3390/molecules27020416

**Published:** 2022-01-10

**Authors:** Mostafa El Khomsi, Hamada Imtara, Mohammed Kara, Anouar Hmamou, Amine Assouguem, Brahim Bourkhiss, Mahmoud Tarayrah, Mashail N. AlZain, Nurah M. Alzamel, Omar Noman, Driss Hmouni

**Affiliations:** 1 Natural Resources and Sustainable Development Laboratory, Department of Biology, Faculty of Sciences, Ibn Tofail University, B.P. 133, Kenitra 14000, Morocco; Hmouni.driss@uit.ac.ma; 2 Faculty of Arts and Sciences, Arab American University Palestine, P.O. Box 240, Jenin 44862, Palestine; 3 Laboratory of Biotechnology, Conservation and Valorisation of Naturals Resources (LBCVNR), Faculty of Sciences Dhar El Mehraz, Sidi Mohamed Ben Abdellah University, B.P. 1796 Atlas, Fez 30000, Morocco; Mohammed.kara@usmba.ac.ma; 4 Laboratory of Engineering, Molecular Organometallic Materials and Environment, Faculty of Sciences Dhar El Mehraz, Sidi Mohamed Ben Abdellah University, B.P. 1796 Atlas, Fez 30000, Morocco; Anouar.Hmamou@usmb.ac.ma; 5 Laboratory of Functional Ecology and Environment, Faculty of Sciences and Technology, Sidi Mohamed Ben Abdellah University, P.O. Box 2202 Imouzzer Street, Fez 30000, Morocco; Assougam@gmail.com; 6 Laboratory of Animal Plant Production and Agro-Industry, Department of Biology, Faculty of Sciences, Ibn Tofail University, B.P. 133, Kenitra 14000, Morocco; Brahim.Bourkhiss@uit.ac.ma; 7 Groupe Hospitalier Cochin-Port Royal, Faculity of Medicine, Institut Cochin, Paris University, CNRS, IN-SERM, 75000 Paris, France; Mahmoud.tarayrah@hotmail.com; 8 Department of Biology, College of Sciences, Princess Nourah Bint Abdulrahman University, P.O. Box 84428, Riyadh 11671, Saudi Arabia; mnalzain@pnu.edu.sa; 9 Department of Biology, College of Science and Humanities, Shaqra University, Shaqra 11961, Saudi Arabia; nalzamel@su.edu.sa; 10 Department of Pharmacognosy, College of Pharmacy, King Saud University, Riyadh 11451, Saudi Arabia; onoman20@gmail.com

**Keywords:** *Anchusa italica* Retz, polyphenols, flavonoids, antioxidant, antibacterial

## Abstract

*Anchusa italica* Retz has been used for a long time in phytotherapy. The aim of the present study was to determine the antioxidant and antibacterial activities of extracts from the leaves and roots of *Anchusa italica* Retz. We first determined the content of phenolic compounds and flavonoids using Folin–Ciocalteu reagents and aluminum chloride (AlCl_3_). The antioxidant activity was determined using three methods: reducing power (FRAP), 2.2-diphenyl-1-picrylhydrazyl (DPPH), total antioxidant capacity (TAC). The antimicrobial activity was investigated against four strains of *Escherichia coli,* two strains of *Klebsiella pneumoniae* and coagulase-negative *Staphylococcus,* and one fungal strain of *Candida albicans.* The results showed that the root extract was rich in polyphenols (43.29 mg GAE/g extract), while the leave extract was rich in flavonoids (28.88 mg QE/g extract). The FRAP assay showed a strong iron reduction capacity for the root extract (IC_50_ of 0.11 µg/mL) in comparison to ascorbic acid (IC_50_ of 0.121 µg/mL). The DPPH test determined an IC_50_ of 0.11 µg/mL for the root extract and an IC_50_ of 0.14 µg/mL for the leaf extract. These values are low compared to those for ascorbic acid (IC_50_ of 0.16 µg/mL) and BHT (IC_50_ 0.20 µg/mL). The TAC values of the leaf and root extracts were 0.51 and 0.98 mg AAE/g extract, respectively. In vitro, the extract showed inhibitory activity against all strains studied, with diameters of zones of inhibition in the range of 11.00–16.00 mm for the root extract and 11.67–14.33 mm for the leaf extract. The minimum inhibitory concentration was recorded for the leaf extract against *E. coli* (ATB:57), corresponding to 5 mg/mL. Overall, this research indicates that the extracts of *Anchusa italica* Retz roots and leaves exert significant antioxidant and antibacterial activities, probably because of the high content of flavonoids and polyphenols.

## 1. Introduction

Aromatic and medicinal plants have been traditionally used in phytotherapy [1,2]. Plants are a rich reservoir of active molecules that are used in the production of medicines around the world [3,4]. They contain a diversity of phytochemical compounds (polyphenols, flavonoids, tannins, terpenes, etc.) [5]. These phytochemicals are responsible for the biological activities of plants, including their antifungal, antioxidant, and antibacterial properties [6] *Anchusa italica* Retz is a perennial plant bearing flowers [7]. Kurds used this plant in food preparation; it was also used as an antitussive, depurative, diuretic, and anti-inflammatory. The flower is harvested and dried for further use. The plant is rich in the alkaloid cynoglossine, which is known for its carcinogenic and paralyzing effects [8]. The flowers of the plant have been traditionally used as a tonic for children and also lower heart rate [9]. This plant is also used to treat cerebrovascular and cardiovascular diseases and diabetes [10,11]. Many studies have shown its anti-inflammatory, neuroprotective and antioxidants activities [4,12]. Plants are an important source of phenolic compounds, which are known for their antibacterial properties and can have applications as natural preservatives either in the food field or in the cosmetic field [4,12,13,14]. Previous chemical studies have shown that *Anchusa italic* Retz is rich in polyphenols, flavonoids, saponins, tannins, vitamin E [15,16,17,18]. The seeds of the plant are rich in saturated fatty acids, unsaturated fatty acids, and alkaloids [19,20,21,22]. However, the antioxidant and antimicrobial activities of extracts of *Anchusa italic* Retz have not been studied. In Morocco, to our knowledge, there are no studies carried out on this plant.

The aim of this research was to determine the antioxidant and antibacterial activiies of extracts of the plant *Anchusa italica* Retz.

## 2. Results and Discussion

### 2.1. Phenolic and Flavonoids Content

**Figure 1:**
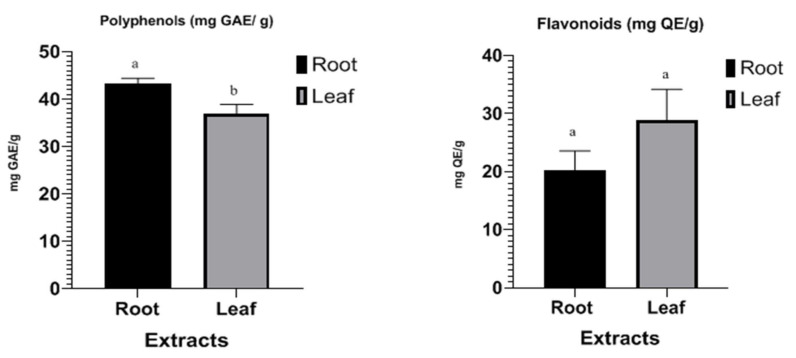
Histogram of polyphenol and flavonoid content of extracts of Anchusa italic Retz. Sta-tistically significant differences between the means are indicated by a and b (*p* < 0.05).

Polyphenol levels were obtained from the linear regression equation of the gallic acid calibration curve y = 0.0056x + 0.0063 and *R*^2^ = 0.9981 and are expressed as milligrams of gallic acid equivalent per gram of extract (mg GAE/g extract). The results indicated a statistically significant difference (*p* < 0.05) between the average phenol contents of two plants extracts, as shown in Figure 1. The phenolic content of the roots extracts was 43.29 ± 1.12 mg GAE/g extract, while the polyphenol content in the leaves was 36.9 mg GAE/g extract.

The polyphenols yields were lower than those found by another study [21]. Other research found that the total polyphenol content of *Anchusa italica* was 16.2 in a methanolic extract and 12.3 in an aqueous extract (gallic acid equivalents per g dry weight) [22]. This amount varies quantitatively and qualitatively from one plant to another, and this can be attributed to meteorological and petrographic changes, the harvesting season, and the vegetative stage of the plant, as well as the extraction methods used [23,24].

The flavonoids content was determined from the calibration curve of quercetin: y = 0.0019x + 0.0558 and *R*^2^ = 0.9844 and expressed in mg EQ/g extract. Our results indicated that the flavonoid content in the roots was 20.29 ± 3.29 mg EQ/g extract and that in the leaves was 28.88 ± 5.28 mg EQ/g extract (Figure 1). Previous studies found that phenolic compounds, including flavonoids, possess strong antioxidant activity and exert health benefits [25].

### 2.2. Antioxidant Activity

#### 2.2.1. Reducing Power Test

The IC_50_ values in Table 1 indicated a statistically significant difference (*p* < 0.05) between the antioxidant activity (FRAP) of the two plant extracts and that of the natural antioxidants ascorbic acid and BHT. The root extract showed a significantly superior antioxidant activity (0.11 ± 0.01 µg/mL) compared to the leaf extract (1.44 ± 0.06 µg/mL) and the reference ascorbic acid (0.12 ± 0.01 µg/mL), but a lower antioxidant activity compared to BHT (0.034 ± 0.00 µg/mL). This indicates that the extracts of *Anchusa italica* have important antioxidant characteristics. Metals ions are necessary for the functioning of biochemical and physiological cellular processes but, at the same time, may cause lipid peroxidation, tissue damage, and oxidative stress when their levels re not regulated [26,27].

Previous research has demonstrated that various flavonoids have antioxidant properties. They can chelate metal ions, the catalysts of the Fenton reaction [28]. Polyphenolic compounds are powerful reducers, and their presence in the examined extracts could have contributed to the FRAP [29].

#### 2.2.2. Scavenging of the Free Radical DPPH

In the DPPH assay, the IC_50_ value of the root extract of *Anchusa italica* Retz was 0.11 ± 0.00 µg/mL and that of the leaf extract was 0.14 ± 0.01 µg/mL (Table 2). The DPPH IC_50_ value for vitamin C was approximately 0.16 ± 0.01 µg/mL, and those for BHT and quercetin were 0.2 ± 0.00 µg/mL and 0.05 ± 0.00 µg/mL, respectively. These results indicate that the radical scavenging capacity of the root extract of *Anchusa italica* is superior to those of vitamin C, BHT, and the leaf extract, but inferior to that of the natural antioxidant quercetin. The statistical analysis of the antioxidant test for the two plant extracts showed a statistically significant differences (*p* < 0.05), as can be seen in Table 2.

Previous studies reported that the IC_50_ value of the free radical scavenging activity on DPPH for *Anchusa italica* Retz was 84 µg/mL [21]. This supports our finding that our extracts are powerful antioxidants. Previous studies indicated that butanol present in *Anchusa italica* and two of triterpene compounds isolated by Kuruüzüm–Uz et al. [30] have strong antioxidant activity against DPPH.

#### 2.2.3. Total Antioxidant Capacity 

The total antioxidant capacity was measured using ascorbic acid at different concentration to obtain an ascorbic acid calibration curve; we expressed the antioxidant capacity of the extracts in milligrams of ascorbic acid equivalent per gram of sample (mg AAE/g extract).

The root extract of the studied plant showed a very strong total antioxidant capacity of 0.98 ± 0.28 mg AAE/g extract with respect to the leaf extract, which showed a total antioxidant activity of about 0.51 ± 0.01 mg AAE/g extract (Table 3).

Recent studies have shown that phenolic compounds, in particular flavonoids, are powerful antioxidants [31]. The antioxidant activity of flavonoids results from the elimination, chelation, and scavenging of free radicals, the inhibition of oxidases, and the chelation of iron ions [32]. HPLC–MS chromatographic analysis showed that the plant *Anchusa italica* is rich in flavonoids including rutin, quercetin, kaempferol, naringenin, hesperidin [17]. Other studies have shown that rutin exhibited antioxidant activity in all three antioxidant tests, i.e., DPPH radical scavenging, reducing power, total antioxidant activity; our results confirm these previous findings [33].

The antioxidant activity of *Anchusa* spp. is well demonstrated in the literature. In fact, *Anchusa azurea* and *Anchusa officinalis* have shown antioxidant activity as DPPH radical scavengers [34,35].

### 2.3. Antimicrobial Studies

#### Determination of the Antimicrobial Activity of the Extracts

Antimicrobial testing of leaves and roots samples of *A. italica* Retz against selected bacterial strains was carried out by using the solid medium disk and microdilution assay. The inhibition zones and the minimum inhibitory concentration of the two extracts are presented in Table 4 and Table 5.

As far as we know, the antimicrobial activity of extracts from the roots and leaves of *Anchusa italica* Retz has not been studied. As per the results of the diameter of inhibition zones (DIZ) presented in Table 4, the extracts showed moderate antibacterial activity against all eight strains studied (*E. coli* (ATB: 97) BGM, *E. coli* (ATB: 57) B6N, *E. coli* (ESBL), *E. coli* sensible, *K. pneumoniae* (ESBL-KP), *K. pneumoniae* sensible, coagulase-negative *staphylococci* and *C. albicans*), with diameters of inhibition zones ranging from 11 to 16 mm. The largest zones of inhibition were found for coagulase-negative *staphylococci* (16.00 ± 1.00 mm) in the presence of the root extract and the leaf extract (13.67 ± 1.15 mm), followed by *E. coli* (ATB: 97) BGM, with an inhibition zone for the root extract of 15.00 ± 1.00 mm and for the leaf extract of 12.33 ± 1.15 mm, and then by *E. coli* sensible, with inhibition diameters for the root and leaf extracts of 13.67 ± 0.58 mm and 14.33 ± 0.58 mm, respectively, followed by *C. albicans*, *E. coli* (ATB: 57) B6N, *E. coli* (ESBL), *K. pneumoniae*, and lastly *K. pneumoniae* (ESBL-KP).

A shown in Table 5, we found that only 5 mg/mL of leaf extracts was sufficient to stop the growth of *E. coli* (ATB: 57) B6N, while 10.00 mg/mL extracts was needed to stop the growth of *E. coli* (ATB: 97), coagulase-negative *staphylococci*, *E. coli* (ESBL), and *E. coli* sensible. On the other hand, *C. albicans*, *K. pneumoniae* (ESBL-KP), and *K. pneumoniae* sensible (KPS) were inhibited by the leaf and root extracts with MIC of 20.00 mg/mL.

Previous studies have shown that the extracts of *Anchusa italica* Retz showed a significant antibacterial activity against *E. coli*, *Bacillus* sp., *S. aureus*, *P. Aeruginosa*) [3].

The most studied plants seem to contain compounds of phenolic nature, especially essential oils, flavonoids, and terpenoids. These compounds have been known for their antimicrobial activity [36]. The antibacterial properties of phenolic compounds have been well demonstrated in other previous studies [37]. HPLC chromatographic analysis showed that the plant *Anchusa italica* is rich in caffeic acid, rutin and astragaline [38]. The antibacterial activity of caffeic acid has been well demonstrated in previous research [37]. Phenolic compounds exert their antimicrobial activity at the cellular level either by modifying the rigidity of the cell wall, the permeability of the cell membrane, or various intracellular functions induced through the formation of hydrogen bonds with enzymes [39,40,41,42].

Previous research has shown that *Anchusa* spp. have antibacterial activity. In fact, the extract of aerial parts of *Anchusa azurea* showed an inhibitory effect on 11 bacteria, including *Escherichia coli, Klebsiella pneumonia*, and *Staphylococcus aureus* [40]. On the other hand, *Anchusa strigosa* has shown strong antibacterial activity against Gram-negative and Gram-positive bacterial strains [12].

## 3. Materials and Methods

### 3.1. Plant Material

The plant parts used in this study, including the roots and leaves of *Anchusa italica* Retz, were collected in the region of MoulayYacoub, Morocco, during the autumn period from November to December 2020. After harvesting, the plant material was washed with water, dried at 30 °C, then crushed and stored in boxes. A hydro–ethanolic extract of the plant was prepared by maceration using 30% distilled water and 70% ethanol for 10% plant powder (mass/volume). After filtration, the filtrate was concentrated by evaporation of the solvent, using a rotary evaporator under partial vacuum at 40 °C, and the dry extract was stored until further use.

### 3.2. Determination of the Phenolic Content

The phenolic content was determined according to the Folin–Ciocalteu protocol with some modifications by using the gallic acid standard [41]. In this test, 0.2 mL of Folin–Ciocalteu reagent was added to 4 mL of 2% Na_2_CO_3_ and 0.2 mL of plant extracts. After half an hour, the determination of the absorbance of the solution was carried out using a spectrophotometer at 760 nm, and the phenolic content was calculated using the equation of the calibration range established with gallic acid, i.e., y = 0.0001x + 0.0627 and *R*^2^ = 0.8694.

### 3.3. Determination of the Flavonoid Content 

The quantification of flavonoids was carried out according to the following protocol [42], with slight modifications. We added 2 mL of the extract to 0.2 mL of 10% AlCl_3_, 0.2 mL of 1 M potassium acetate, and 7.6 mL of ethanol. After 40 min, the absorbance was determined by spectrophotometry at 430 nm, and the flavonoid content was determined using the equation of the calibration range established with quercetin, i.e., y = 0.0019x + 0.0558 and *R*^2^ = 0.9844.

### 3.4. Antioxidant Tests 

#### 3.4.1. Reducing Power Test 

The reducing power test was performed according to the following protocol [43]. We added 750 µL of ferricyanide of potassium [K_3_Fe(CN)_6_] 1% to 750 µL phosphate buffer (0.2 M: pH = 6.6) and 1.5 mL of *Anchusa italica* samples previously prepared in methanol at a concentration of 10 mg/mL. The mixtures were incubated in a water bath for 20 min at 50 °C. Then, 150 µL of FeCl_3_ (1%), 750 µL of aqueous solution of TAC (10%), and 750 µL of distilled water were added to the prepared solution. The absorbance of the solution was determined using a spectrophotometer at 700 nm, in comparison to a solution containing all the above components except the plants extracts (negative control). The result was expressed as 50% effective concentration (IC_50_), i.e., the concentration of antioxidant needed to have an optical density of 0.5.

#### 3.4.2. Free Radical Scavenging Capacity 

The determination of (2,2-diphenyl-1-picrylhydrazyl) was performed according to the following protocol [44]. We mixed 200 µL of each series dilution of the two extracts with 750 µL of DPPH (0.004%). After half an hour in the dark, the absorbance of the mixtures was determined at 517 nm. The following equation was used to calculate the percent inhibition (PI):

PI(%) = ((A_0_ − A)/A_0_) × 100.

A_0_: Absorbance of the solution (DPPH) without the extracts.
A: Absorbance of the solution (DPPH) with the extracts.

#### 3.4.3. Total Antioxidant Capacity

The antioxidant test (TAC) was determined by the following protocol [45]. We added 1 mL of a prepared solution (4 mM ammonium molybdate, 28 mM sodium phosphate, 0.6 M acid sulphuric) to 25 µL of the extract studied. Then, the reaction mixture was incubated in a water bath for half hour at 95 °C. The absorbance of the solution was determined at 695 nm using a negative control containing 25 µL of methanol in place of the extracts. TAC was expressed as mg AAE/g extract. A standard curve was prepared using ascorbic acid.

### 3.5. Antimicrobial Activity of Anchusa italica Retz

The antimicrobial activity was investigated against eight bacterial strains: *Escherichia coli* (ATB: 57) B6N, *Escherichia coli* (ATB: 97) BGM, *Escherichia coli* sensible, *Klebsiella pneumoniae* (ESBL-KP), *Klebsiella pneumoniae* sensible, and coagulase-negative *staphylococci* (CoNS) and one yeast strain, i.e., *Candida albicans*. The tested microorganisms were obtained from the microbiology laboratory FMP-Fez. Bacterial suspensions were obtained by picking colonies from 24 h cultures. The prepared cultures were stored in MH agar at 4 °C. In a sterile solution (0.9% NaCl), the colonies were suspended and then shaken for 15 s. The density was set at 0.5 turbidity (equivalent to 1–5 × 10^8^ CFU/mL) [46].

#### 3.5.1. Agar Disk Diffusion

This test is based on the following method described by Sadiq et al. [44], with a slight modification. MH agar plates were prepared from standardized bacterial suspensions (10^8^ CFU/mL) and inoculated by swabbing. Then, Whatman paper discs (6 mm) were placed on the surface of the pre-inoculated agar. Then, each disk was soaked in 20 µL of 50 mg/mL extract of *Anchusa italica* diluted in dimethyl sulfoxide (DMSO), at a concentration of 4 mg/L. A disk containing DMSO was placed in the middle of each Petri dish and was used as a negative control. Afterwards, the plates were incubated for 24 h at 37 °C, and the diameters of the inhibition zones were measured. The studied bacteria were classified as resistant or sensitive to the extracts based on the diameter of the inhibition zones (DIZ) as follows [47]:

- Not sensitive: DIZ was lower than 8 mm
- Sensitive: DIZ was between 9 and 14 mm
- Very sensitive: DIZ was between 15 and 19 mm
- Extremely sensitive: DIZ was superior then 20 mm

#### 3.5.2. Minimum Inhibitory Concentration (MIC)

The determination of MIC values was performed by microdilution tests in 96-well plates using NCCLS standards [48], with slight modifications. Extracts from the roots and leaves of *Anchusa italica* were prepared in sterile hemolysis tubes using DMSO. The final concentrations of the extracts in the wells were obtained by successive 1:1 dilutions in a mixture of Mueller Hinton (MH) broth, reaching values between 0.039 and 20 mg/mL. Then, 50 µL of microbial suspensions was mixed with 50 µL of MH broth, and 50 µL of extract solutions at different concentrations was added to determine the MIC values. After incubating the plates for 18 h at 37 °C, 40 µL of 0.5% triphenyl tetrazolium chloride was added to each well. The MIC value was determined in the basis of the lowest concentration that did not produce a red color [46].

### 3.6. Statistical Analysis

Values in this study are presented as means ± standard deviations. Student’s t-test and one-way ANOVA were used to analyze the differences between the experimental data. The significance of changes in DPPH and FRAP was determined by the Tuckey test as a post hoc test. Significance for all tests was established at *p* < 0.05 using Minitab 19.1 software (LLC, New York, NY, USA).

## 4. Conclusions

The results of this work showed that the extracts of leaves and roots of *Anchusa italica* Retz are rich in compounds with antioxidant and antimicrobial properties. More study is needed to support the antioxidant and antibacterial activities of *Anchusa italica* Retz extracts, which can have several applications in phytotherapy and pharmaceutical therapy to treat human diseases and as preservatives for raw foods. Based on this study, further research is needed for the purification and chemical identification of active molecules with biological properties contained in these extracts. 

## Figures and Tables

**Table 1:** IC_50_ (50% effective concentration) of ascorbic acid, BHT, and extracts of *Anchusa italica*, using the FRAP method.

Extract	IC_50_ (µg/mL)
Roots	0.11 ^bc^ ± 0.01
Leaves	1.44 ^a^ ± 0.06
Ascorbic acid	0.12 ^b^ ± 0.01
BHT	0.03 ^c^ ± 0.00

Statistically significant differences between the means are indicated by ^a^, ^b^, and ^c^ (*p* < 0.05).

**Table 2:** Values IC_50_ from the DPPH test.

Extract	IC_50_ (µg/mL)
Roots	0.11 ^d^ ± 0.00
Leaves	0.14 ^c^ ± 0.01
Ascorbic acid	0.16 ^b^ ± 0.01
BHT	0.20 ^a^ ± 0.00
Quercetin	0.05 ^e^ ± 0.00

Statistically significant differences between the means are indicated by ^a^, ^b^, ^c^, ^d^ and ^e^ (*p*< 0.05). Values in the same column followed by the same letter are not significant different (*p* < 0.05) by the Tukey’s multiple range test.

**Table 3:** Total antioxidant capacity of the two extracts of *A. italica* Retz.

Extract	TAC (mg AAE/g Extract)
Roots	0.98 ^a^ ± 0.28
Leaves	0.51 ^b^ ± 0.01

Statistically significant differences between the means are indicated by ^a^ and ^b^ (*p* < 0.05).

**Table 4:** Diameter of inhibition zones of *Anchusa italica* Retz extracts.

Bacteria Strains	Diameter of Inhibition Zones (mm)	
	Root Extract	Leaf Extract
*E. coli (ATB: 57) B6N*	13.67 ^a^ ± 0.58	11.67 ^b^ ± 0.58
*E. coli (ATB: 97) BGM*	15.00 ^a^ ± 1.00	12.33 ^b^ ± 1.15
*K. pnemonia (ESBL-KP)*	11.33 ^b^ ± 0.58	12.67 ^a^ ± 0.58
*Klebsiella pneumoniae* sensible	11.00 ^b^ ± 1.00	13.67 ^a^ ± 0.58
coagulase-negative *staphylococci*	16.00 ^a^ ± 1.00	13.67 ^a^ ± 1.15
*C. albicans*	12.67 ^a^ ± 0.58	13.67 ^a^ ± 1.15
*E. coli (ESBL)*	12.67 ^a^ ± 0.58	13.33 ^a^ ± 0.58
*E. coli* sensible	13.67 ^a^ ± 0.58	14.33 ^a^ ± 0.58

Statistically significant differences between the means are indicated by ^a^, ^b^, (*p* < 0.05).

**Table 5:** The minimum inhibitory concentration (MIC) of *Anchusa italica* Retz extracts.

Bacteria Strains	Concentration mg/mL	
	Root Extract	Leaf Extract
*E.coli* (ATP: 57) B6N	10.00	05.00
*E. coli* (ATP: 97) BGM	10.00	10.00
*K. pnemoniae (ESBL-KP)*	20.00	20.00
*Klebsiella pneumoniae* sensible	20.00	20.00
coagulase-negative *staphylococci*	10.00	10.00
*C. albicans*	20.00	20.00
*E. coli* (ESBL)	10.00	10.00
*E. coli* sensible	10.00	10.00

## Data Availability

Not applicable.

## Sample Availability

Samples are available from the authors upon reasonable request.
